# Decision Support Techniques for determination of the causal relationship between health problems of workers and their work activities

**DOI:** 10.47626/1679-4435-2023-1099

**Published:** 2024-09-24

**Authors:** Douglas de Almeida Martins, Dalessandro Soares Vianna, Marcilene de Fatima Dianin Vianna

**Affiliations:** 1 Division of Health Forensics, Universidade Federal Fluminense (UFF), Rio das Ostras, RJ, Brazil; 2 Institute of Science and Technology, UFF, Rio das Ostras, RJ, Brazil

**Keywords:** occupational health, occupational medicine, occupational diseases, decision support techniques, decision making, saúde do trabalhador, medicina do trabalho, doenças profissionais, técnicas de apoio para a decisão, tomada de decisões

## Abstract

Reflecting on the complexity and impacts of determination of the causal relationship between health problems of workers and the exercise of their work activities, there is a need to learn about scientific articles that expose techniques to determine this type of causal relationship. There is also a need to reveal whether any article exposes multicriteria decision analysis technique. The aim is to quantify the techniques used to determine the causal relationship between health problems of workers and the exercise of their work activities. Bibliometric analysis was performed, searching for articles in Portuguese, Spanish and English. An advanced search was performed on the website of the ministerial journals portal and then on the Gale Academic OneFile, SciVerse Scopus, Scientific Electronic Library Online (SciELO) and PubMed Central collections. In summary, 38 articles were selected from portal, 50 from Gale Academic OneFile, 20 from SciVerse Scopus, 37 from SciELO and 5 from PubMed Central, totaling 150 articles of interest for analysis of their contents. Among these 150 articles, 33.33% addressed the causal relationship between illness and work, 3.33% described some process related to occupational diagnostic investigation and 0.66%, which represents only one article, exhibited a technique to determine this type of causal relationship: the probability of causality in neoplastic diseases. No article described multicriteria decision analysis method as a technique for determine this type of causal relationship. Therefore, there is a need to carry out and disseminate scientific research on methods to help determine a causal relationship between illness and work.

## Abstract (pt)

Ao refletir sobre a complexidade e os impactos do estabelecimento do nexo causal entre o agravo à saúde dos trabalhadores e o exercício de suas atividades laborais, surge a necessidade de conhecer artigos científicos que expõem técnicas para estabelecer esse tipo de nexo. Surge também a necessidade de revelar se algum artigo expõe auxílio multicritério à decisão. O objetivo foi quantificar as técnicas utilizadas no estabelecimento do nexo causal entre o agravo à saúde dos trabalhadores e o exercício de suas atividades laborais. Foi realizada uma análise bibliométrica, buscando artigos em português, espanhol e inglês. Realizou-se uma busca avançada no *site* do portal ministerial de periódicos e, em seguida, nas coleções Gale Academic OneFile, SciVerse Scopus, Scientific Electronic Library Online (SciELO) e PubMed Central. Em síntese, foram selecionados 38 artigos do portal ministerial de periódicos, 50 da Gale Academic OneFile, 20 da SciVerse Scopus, 37 do SciELO e 5 da PubMed Central, totalizando 150 artigos para análise de conteúdo. Entre esses 150 artigos, 33,33% abordavam o nexo causal entre doença e trabalho, 3,33% descreviam algum processo relacionado à investigação diagnóstica ocupacional, e 0,66%, o que representa apenas um artigo, exibia uma técnica para se estabelecer esse tipo de nexo causal: a probabilidade de causalidade em doenças neoplásicas. Nenhum artigo descrevia o auxílio multicritério à decisão como técnica para estabelecer esse tipo de nexo causal. Portanto, nota-se a necessidade da realização e divulgação de pesquisas científicas sobre métodos de auxílio ao estabelecimento de nexo causal entre doença e trabalho.

## INTRODUCTION

The causal relationship between health problems experienced by workers and their work activities is a problem which requires doctors specializing in occupational medicine to analyze various criteria to decide whether or not to recognize the causal relationship. The Conselho Federal de Medicina (CFM, Brazil’s Federal Council of Medicine)^1^ states that, in addition to anamnesis, clinical examination (physical and mental), reports and complementary tests, physicians must consider: I - the current and previous clinical and occupational history, which is decisive in any diagnosis and/or investigation of a causal relationship; II - the investigation of the workplace; III - the investigation of the work organization; IV - epidemiological data; V - scientific literature; VI - the occurrence of clinical or subclinical conditions in workers exposed to similar risks; VII - the identification of physical, chemical, biological, mechanical, stressful, and other hazards; VIII - the testimony and experience of workers; and IX - the knowledge and practices of other disciplines and their professionals, regardless whether they are health-related or not.

In terms of the employment relationship governed by the Consolidação das Leis do Trabalho (CLT, Brazil’s Consolidation of Labor Laws), some relevant losses of a causal relationship that is mistakenly denied can be highlighted: loss of the guaranteed maintenance of the employment contract with the company, for at least 12 months, after the termination of the accident sick leave allowance^2^; the loss of a social security benefit^2^; the lack of deposit in the Fundo de Garantia do Tempo de Serviço (FGTS, Severance Indemnity Fund) paid by the company during the leave due to an accident at work and the payment of the accident sick leave allowance^3^; and a reduced Fator Acidentário de Prevenção (FAP, Accident Prevention Factor)^4^ and, consequently, the contribution rate levied on the company’s payroll.^5^ Furthermore, regardless of the employment relationship, this misunderstanding represents a missed opportunity to improve working conditions and the workplace after an occupational relationship has been recognized.

The causal relationship between disease and work is a complex cognitive decision-making process and depends on the physician’s medical expertise. Methods to assist this process would have great potential for applicability. Reflecting on the complexity and impact of this decision, the need arises to find out about scientific studies that present techniques to determine the causal relationship in occupational diseases. There is also a need to find out whether any scientific articles use multicriteria decision analysis techniques to determine this type of causal relationship. This study aims to quantify the techniques used to determine the causal relationship between workers’ health problems and their work activities.

## METHODS

A comprehensive bibliometric analysis of this topic was conducted by searching for scientific articles in three languages: Portuguese, Spanish, and English. Advanced searches in Portuguese used the following keywords: *nexo laboral, nexo ocupacional,* and *nexo causal trabalho.* In Spanish, the following keywords were used: *nexo laboral* and *nexo causal trabajo.* In English, we used: labour nexus, labor nexus, occupational nexus, and work causal nexus. As a priority, these keywords were found in the title, abstract, and keywords of scientific articles. In databases where searching for these keywords in the title, abstract, or keywords was not possible, we searched for them in the full document. These searches are detailed below to allow for reproducibility, visualization of the scope, and identification of duplicate journals, as the same scientific article can be found in more than one collection and in more than one language.

Advanced searches were conducted on the Coordenação de Aperfeiçoamento de Pessoal de Nível Superior do Ministério da Educação (CAPES/ MEC, Coordination for the Improvement of Higher Education Personnel of Brazil’s Ministry of Education) website, with access by federated academic community via Universidade Federal Fluminense (https://www-periodicos-capes-gov.br.ez24.periodicos.capes.gov. br/), for the period between 1940 and 2021. The CAPES advanced search allows the combination of only two keywords per search. We then made two advanced searches in the two largest virtual journal databases identified by the initial search on the CAPES website: Gale Academic OneFile (Gale) and SciVerse Scopus (Scopus). In addition, an advanced search was made in the Scientific Electronic Library Online (SciELO) and PubMed Central databases, due to the specific nature of the topic, which is strongly associated with occupational medicine. These four collections allow several keywords to be combined per search.

**Figure 1:**
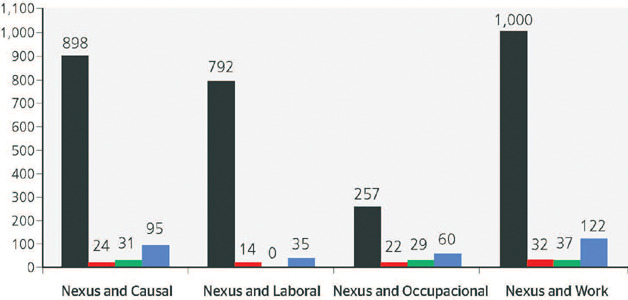
Number of health-related, peer-reviewed articles between 1940 and 2021 using the Coordenação de Aperfeiçoamento de Pessoal de Nível Superior/Ministério da Educação (CAPES/MEC, Coordination for the Improvement of Higher Education Personnel/Ministry of Education and Culture) advanced search in Portuguese. Blue = number of health-related articles; Black = total number of articles; Green = number of occupational health-related articles; Red = number of *saúde do trabalhador*-related articles.

In the advanced search in Portuguese on CAPES, keywords were found anywhere in the article and peer-reviewed scientific articles were selected. A total of 898 articles were found with the words *nexo* and *causal,* 792 with the words *nexo* and *laboral,* 257 with the words *nexo* and *ocupacional* and 1,000 with the words *nexo* and *trabalho.* Figure 1 shows the refinement of this search, highlighting the main health topics classified on the CAPES database: *saúde do trabalhador,* occupational health, and public health. In general, the health-related topic, namely causal relationship between disease and work is little covered in the scientific literature in Portuguese on the subject of *nexo causal.* The two main collections dealing with the subject of causal relationship between disease and work in Portuguese are Gale and Scopus.

**Figure 2:**
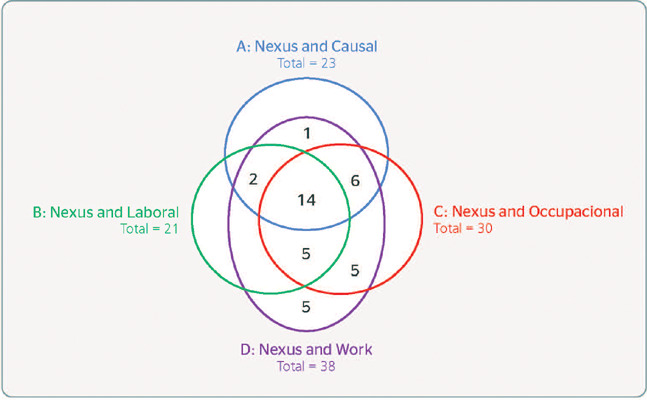
Representation of the sets of keyword pairs found on the Revista Brasileira de Medicina do Trabalho using the Coordenação de Aperfeiçoamento de Pessoal de Nível Superior/Ministério da Educação (CAPES/MEC, Coordination for the Improvement of Higher Education Personnel/Brazil’s Ministry of Education).

The main health-related CAPES journals were Ciência e Saúde Coletiva and Revista Brasileira de Medicina do Trabalho (RBMT) (Table 1). It is important to note that the RBMT has a more specific approach to the subject of causal relationship between disease and work, and all RBMT scientific articles found on CAPES belonged to the Gale collection. Figure 2 shows the unduplicated sets of keyword pairs on CAPES for RBMT. Furthermore, articles from this journal on the subject can also be found in the Scopus and SciELO collections.

**Table 1:** Number of health-related, peer-reviewed articles between 1940 and 2021 found in scientific journals using CAPES/MEC advanced search in Portuguese

Word pairs in any part of the article	Total peer-reviewed articles between 1940 and 2021	Ciência e Saúde Coletiva	Revista Brasileira de Medicina do Trabalho
*Nexo* and *causal*	898	47	23
*Nexo* and *laboral*	792	25	21
*Nexo* and *ocupacional*	257	43	31
*Nexo* and *trabalho*	1,000	80	42

CAPES/MEC = Coordenação de Aperfeiçoamento de Pessoal de Nível Superior/Ministério da Educação (Coordination for the Improvement of Higher Education Personnel/Brazil’s Ministry of Education).

Peer reviewed scientific articles were also selected using the CAPES advanced search in Spanish, looking for the keywords anywhere in the article. A total of 792 articles were found with the keywords *nexo* and *laboral,* and 2,638 with the keywords *nexo* and *trabajo.* We also found that, in general, health-related topics (causal relationship between disease and work) are little covered in the scientific literature in Spanish on the subject of causal relationship. We also found that the two main collections dealing with the subject of causal relationship between disease and work in Spanish are Gale and Scopus. The main CAPES health-related scientific journals were Ciência e Saúde Coletiva and Revista de Estudios Sociales. This analysis failed to identify any Spanish-language journals with a more specific approach to the topic of causal relationship between disease and work.

**Figure 3:**
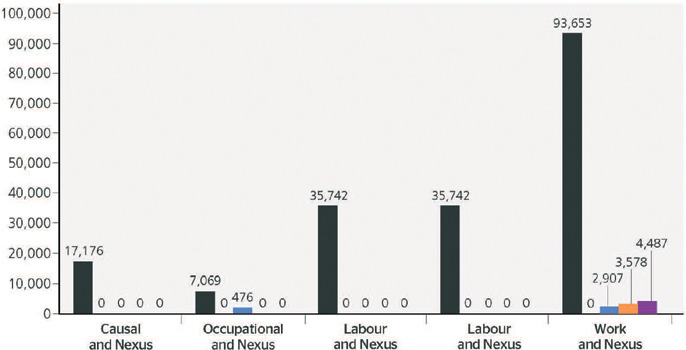
Number of peer-reviewed health-related articles between 1940 and 2021 using the Coordenação de Aperfeiçoamento de Pessoal de Nível Superior/Ministério da Educação (CAPES/MEC, Coordination for the Improvement of Higher Education Personnel/Ministry of Education) advanced search in English. Blue = number of public health articles; orange = number of social welfare and social work articles; black = total number of articles; purple = number of medicine articles; green = number of occupational health articles.

The CAPES advanced search in English, looking for keywords anywhere in the article, also included peer-reviewed scientific articles. It found 17,176 articles with the words causal and nexus, 7,069 with the words occupational and nexus, 35,742 with the words labour and nexus, 35,742 with the words labor and nexus, and 93,653 with the words work and nexus. Figure 3 shows the refinement of this search, highlighting the main CAPES health-related topics: social welfare and social work, medicine and public health. We also found that, in general, health-related topics (causal relationship between disease and work) are little covered in the English scientific literature on the subject of causal relationship. Furthermore, the two main collections addressing the topic causal relationship between disease and work in English are Gale and Scopus. The main CAPES-ranked health-related scientific journals are: International Organization, Environmental Health Perspectives, Labor Studies Journal and International Journal of Environmental Research and Public Health. This analysis also failed to identify any English-language journals that presented a more specific approach to the subject of the causal relationship between disease and work.

The Gale advanced search found 100 full-text documents, using the following keywords throughout the document: *nexo laboral,* or *nexo ocupacional,* or *nexo causal trabalho,* or *nexo causal trabajo,* or labour nexus, or labor nexus, or occupational nexus, or work causal nexus. Of these 100 documents, 96 were peer-reviewed. Of the 96, 76 were scientific articles. Among these 76, eight generated duplicates with the CAPES advanced search with the keywords *nexo* and *causal* and belonged to the RBMT. In addition, 18 articles were related to Law, with no content of interest to this dissertation. Of the 50 unduplicated scientific articles found on the Gale database, two also belonged to the RBMT.

The Scopus advanced search found 126 documents using the following keywords in the title, abstract, or keywords of the journals: *nexo laboral,* or *nexo ocupacional,* or *nexo causal trabalho,* or *nexo causal trabajo,* or labour nexus, or labor nexus, or occupational nexus, or work causal nexus. Of these 126 documents, 99 were scientific articles. We obtained 23 scientific articles after narrowing down the results of this last search with the following sources as inclusion criteria: Revista Brasileira de Medicina do Trabalho; Psicologia e Sociedade; Arquivos Brasileiros de Psicologia; Cadernos de Saúde Pública/Ministério da Saúde, Fundação Oswaldo Cruz, Escola Nacional De Saúde Pública;

Criminal Justice Review; Espacios; Giornale Italiano di Medicina del Lavoro ed Ergonomia; International Labor Review; International Review of Sociology; International Journal of Environmental Research and Public Health; International Journal of Law and Psychiatry; International Journal of Psychoanalysis; Journal of Health Psychology; Journal of Studies on Alcohol; Procedia Manufacturing; Revista Brasileira de Neurologia e Psiquiatria; Revue Française de Sociologie; Social Behavior and Personality; and Sociology. Of these 23 articles, 3 were duplicates in the CAPES advanced search with the keywords *nexo* and *causal* and belonged to the RBMT.

The SciELO advanced search found 36 full-text scientific articles with no duplicates, using the following keywords: *nexo laboral,* or *nexo ocupacional,* or *nexo causal trabalho,* or *nexo causal trabajo,* or labour nexus, or labor nexus, or occupational nexus, or work causal nexus. The PubMed Central advanced search found five open access scientific articles with the keyword nexus in the abstracts of the journals. These articles are from the Centers for Disease Control and Prevention, which includes the National Institute for Occupational Safety and Health.

**Figure 4:**
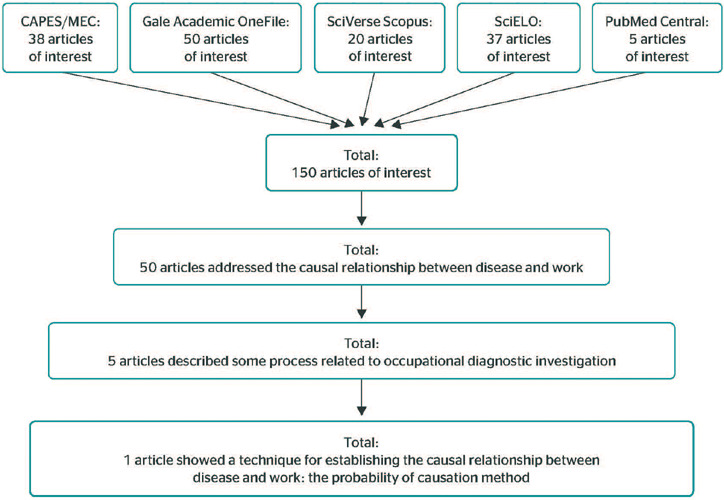
Summary of advanced searches. CAPES/MEC = Coordenação de Aperfeiçoamento de Pessoal de Nível Superior/Ministério da Educação - Coordination for the Improvement of Higher Education Personnel/Brazil’s Ministry of Education; SciELO = Scientific Electronic Library Online.

The quantitative data analysis included 38 articles from CAPES (which also belong to Gale), 50 from Gale, 20 from Scopus, 37 from SciELO and 5 from PubMed Central, totaling 150 scientific articles of interest for content analysis (Figure 4).

Following this analysis, in addition to duplicates in different collections, the following contents were defined as exclusion criteria in this study: migration, suicide, violence at work, sociology, economics, service provision, law, forensic medicine, philosophy, prevention, education, child labour, molecular biology, psychoanalysis, imaging exams, ergonomics, accident prevention factor, social security benefits, administration, medical clinic, dentistry, book review, occupational health medical control program, absenteeism, profile of occupational physicians, dysthanasia, history, and organizational psychology.

## RESULTS

Among the 150 scientific articles of interest, 50 addressed the causal relationship between disease and work. Of the 50 articles, 5 described some process related to occupational diagnostic investigation. Chart 1 shows these 5 articles, and summarizes their content analysis.

**Chart 1:** Summary of the analysis of the five most relevant scientific articles describing a process related to occupational diagnostic investigation

Journal title (year/collection)	Title of article (author)	Summary of content analysis
Revista Brasileira de Medicina do Trabalho (2016/Gale Academic OneFile)	Perda auditiva induzida por ruído ou complicação da otite média crônica? (Oliveira^6^)	It describes a technical-scientific analysis on the characterization of noise-induced hearing loss and its occupational nexus.
Giornale Italiano di Medicina del Lavoro ed Ergonomia (2017/SciVerse Scopus)	AIRM recommendations for the application of probability of causation method (Moccaldi^7^)	It describes the Probability of Causation method, a tool used to identify the causal relationship in compensation claims in the insurance sector, for which it was initially used, and to resolve legal disputes, both civil and criminal, in countries such as the United States of America, the United Kingdom, Japan and South Korea. This method, which uses the foundations of epidemiology, has recently been proposed as an aid for occupational physicians in the case of suspected neoplastic occupational diseases, thanks to AIRM.
Psicologia: Ciência e Profissão (2018/SciELO)	Trabalho e Adoecimento Psicossomático: Reflexões sobre o Problema do Nexo Causal (Rabelo et al.^8^)	It describes a reflection on the relationship between pathogenic factors present in work organization and the development of psychosomatic diseases in workers.
Ciência & Saúde Coletiva (2019/Gale Academic OneFile)	Em busca do reconhecimento do distúrbio de voz como doença relacionada ao trabalho: movimento histórico-político (Masson et al.^10^)	It describes a technical-scientific analysis of the historical advances in the characterization of voice disorder and its work-related nexus.
Revista Gaceta Laboral (2020/Gale Academic OneFile)	La infeccion por SARS-CoV-2 (COVD-19): ¿enfermedad ocupacional o un accidente de trabajo? (Cuauro^11^)	It describes a legal documentary investigation to determine whether COVID-19 can be considered an occupational disease or an occupational accident, when the worker is exposed to contagion by the SARS-CoV-2 due to their work activity.

SciELO = Scientific Electronic Library Online; AIRM = Associazione Italiana di Radioprotezione Medica.

Thus, the databases searched (Gale, Scopus, SciELO and PubMed Central, which are strongly associated with occupational medicine, published 150 scientific articles of interest between 1940 and 2021. Of these, 33.33% of the articles addressed the causal relationship between disease and work. However, 3.33% of these articles covered the process to determine the causal relationship in occupational diseases, and 0.66% (1) showed a technique for establishing this type of causal relationship.

## DISCUSSION

Oliveira^6^ describes a technical-scientific analysis of the characterization of noise-induced hearing loss and its causal relationship with work. So as to confidently conclude that there was no causal relationship between the health problem and the work activities, the author analyzed relevant technical criteria: medical history, physical examination, the attending physician’s medical record, complementary examination, clinical-occupational history, study of the workplace, study of work organization, scientific literature and study of occupational risks.

Moccaldi^7^ describes the probability of causation method, a tool used to identify the causal relationship in compensation claims in the insurance sector, the purpose for which it was initially used, and to resolve legal disputes, both civil and criminal, in countries such as the United States of America, the United Kingdom, Japan, and South Korea. This method, which uses the foundations of epidemiology, has recently been proposed by the Associazione Italiana di Radioprotezione Medica (AIRM) as an aid for occupational physicians in the case of suspected neoplastic occupational diseases.

In a case of psychological expertise in the labor court concerning a worker in the tele-service sector with an alleged depressive episode associated with a somatization disorder and the development of an autoimmune disease (systemic lupus erythematosus), Rabelo et al.^8^ wrote a reflection on the relationship between pathogenic factors present in the work organization and the development of psychosomatic diseases in workers. These authors demonstrated the importance of the testimonies of other workers in the same company, epidemiological studies and the knowledge of a different discipline, Psychology, in the process of investigating the causal relationship between disease and work. When reporting the case of Chalons,^9^ they also demonstrated the relevance of studying the occurrences of clinical or subclinical conditions in workers exposed to similar risks in this causality analysis. Rabelo et al.^8^ also highlighted other technical criteria: medical history, physical examination, complementary tests, clinical-occupational history, study of the workplace, study of work organization and scientific literature.

In a narrative review comprising three axes of analysis, Masson et al.^10^ describe a technical-scientific analysis of the historical advances in the characterization of voice disorders and their relationship with work, a legal-institutional analysis and a political-professional analysis. Masson et al.^10^ emphasized the participation of epidemiological studies and the knowledge of a third discipline, Speech and Hearing Therapy, in the process of recognizing voice disorders as work-related diseases. In this narrative, they also highlighted other technical criteria: the medical anamnesis, the physical examination, the complementary examination, the clinical-occupational history, the study of the workplace, the study of work organization and the study of occupational risks.

Cuauro^11^ highlights the importance of studying the workplace and occupational hazards to establish the causal relationship between the disease and work. Cuauro^11^ concludes that, in Venezuela, COVID-19 can be considered an occupational disease when the worker is exposed to being infected with the SARS-CoV-2 as a result of their work activity, even if they comply with biosafety regulations, in accordance with article 70 of the Ley Orgánica de Prevención, Condiciones y Medio Ambiente de Trabajo:

occupational disease is understood to be pathological states acquired or aggravated as a result of work or exposure to the environment in which the worker is obliged to work, such as those attributable to the action of physical and mechanical agents, nonergonomic, meteorological, chemical conditions, biological agents, psychosocial and emotional factors, manifested by an organic lesion, enzymatic, or biochemical disorders, functional disorders or mental imbalance, temporary or permanent (author’s translation).

## CONCLUSIONS

A meticulous bibliometric analysis found only one scientific article that sets out a technique for establishing the causal relationship in occupational disease, the probability of causality in neoplastic diseases.^7^ No article presenting multicriteria decision analysis techniques to determine this relationship was found. Multicriteria decision analysis techniques have been used mainly in problems related to production engineering and operational research. It is therefore suggested that scientific research be conducted and disseminated on techniques that help determine a causal relationship between workers’ health problems and their work activities.
